# Outpatient Visits among Older Adults Living Alone in China: Does Health Insurance and City of Residence Matter?

**DOI:** 10.3390/ijerph17124256

**Published:** 2020-06-15

**Authors:** Jianyun Wang, Yaolin Pei, Renyao Zhong, Bei Wu

**Affiliations:** 1 School of Public Administration, East China Normal University, 3663 North Zhongshan Road, Shanghai 200062, China; wangjianyunaaa@126.com; 2 Rory Meyers College of Nursing, New York University, 433 First Avenue, New York, NY 10010, USA; yp22@nyu.edu; 3 NYU Aging Incubator, New York University, 433 First Avenue, New York, NY 10010, USA

**Keywords:** outpatient visits, older adults living alone, health insurance, cities of residence, inequalities

## Abstract

This study aimed to examine the association between health insurance, city of residence, and outpatient visits among older adults living alone in China. A sample of 3173 individuals was derived from “Survey on Older Adults Aged 70 and Above Living Alone in Urban China” in five different cities. Logistic regression models indicated that older adults living alone who had urban employee basic medical insurance, urban resident basic medical insurance, and public medical insurance were more likely to have outpatient visits than those without any health insurance. After controlling the number of chronic diseases, only those with public medical insurance were more likely to have outpatient visits than uninsured older adults. Additionally, older adults who resided in Shanghai and Guangzhou were more likely to have outpatient visits than those in Chengdu, whereas older adults who were in Dalian and Hohhot were less likely to have outpatient visits. To improve the equity of outpatient visits among older adults living alone in China, policy efforts should be made to reduce fragmentation of different health insurance plans, expand the health insurance coverage for older adults, provide programs that consider the needs of this special group of older adults, and reduce the inequality in health resources and health insurance policies across cities.

## 1. Introduction

The number of older adults living alone has grown rapidly in recent years, because of changing living arrangements, weakening filial piety, decreased family size, massive population migration, and longer lifespan of women [1]. A study shows that the proportion of older adults living alone is 12.5% in China [2]. In urban China, the proportion of older adults who live alone or live with spouses only is around 50%, increasing to over 70% in large cities, which is much higher than those in rural areas, which is 37% [3]. Particularly, due to the one-child policy, the number of older adults living alone is also predicted to increase significantly in the foreseeable future.

Living alone may increase the needs of outpatient visits as a result of lacking available family caregivers, inability to depend on others to access medical and health needs [4], or lacking assistance in basic activities of daily living (ADLs) and instrumental activities of daily living (IADLs) [5,6,7]. However, older adults living alone have more difficulties in outpatient visits than those living with others, due to lacking assistance in assessing health status, not receiving encouragement to visit a physician, lack of transportation to physician’s offices, difficulties in navigating the healthcare system, paying medical expenses, and so forth [8,9,10]. Therefore, older adults living alone may delay or non-receipt of outpatient visits when they have health problems, which may result in increased complications, or even mortality [11,12,13]. It is important to study outpatient visits because it helps patients to reduce the need for emergency visits and hospitalization, which could reduce overall healthcare costs. In addition, it is an important measure for health promotion, disease prevention and early diagnosis of chronic disease, and improvement in the quality of life for older adults living alone.

Access to health services includes two main important factors: affordability and availability from both the individual and regional perspectives [14]. The affordability of outpatient visits is mainly related to health insurance. Previous studies show that health insurance could improve outpatient visits of older adults [15,16,17,18,19,20]. The availability of outpatient visits is mainly related to the city of residence, which is linked to health care resource allocations, governmental funding and government policies [14]. However, little is known about the influence of different health insurance plans on outpatient visits among older adults living alone in urban areas under China’s unique health insurance system. In addition, few studies explored the association between city of residence and outpatient visits. 

Given the rapid growth of older adults living alone and their greater needs and difficulties in utilization of outpatient service, it is important to understand how health insurance and city of residence are associated with utilization of outpatient visits by the segment of the aging population. Understanding these relationships may provide scientific foundation for the development of effective interventions aimed to promote universal access to health care. Therefore, the present study aimed to examine the association between health insurance, city of residence, and outpatient visits among older adults living alone in urban China. The findings from this study may provide insights in other developing countries, as those countries are also making efforts to promote universal access to health care. 

### 1.1. Background: The Current Health Insurance System in China

For the past two decades, China has made efforts to provide universal health insurance coverage by establishing multiple health insurance plans that are determined by occupation and place of residence (i.e., urban vs. rural) [21]. Currently, there are three health insurance plans among older adults in Chinese urban areas: public medical insurance (only covers retired government employees, implemented in 1998), urban employee basic medical insurance (established in 1998, covers urban retired employees only), and urban resident basic medical insurance (established in 2007, urban residents who are retired as contract workers or have no working history) [8,22,23,24,25]. Furthermore, health insurance plans are managed by city-level local government, the details regarding the utilization and reimbursement of health insurance policies vary across cities [21]. The coverage rate of health insurance system has been over 95% since 2011 [26]. Despite eligibility for health insurance, some older adults are still without any health insurance, especially urban older adults living alone, who are less likely to be aware of the policy and lack of financial resources. A study showed that 33.5% of urban residents were not enrolled in health insurance because of unawareness of the policy, 7.3% did not qualify, and 8% low-income older adults could not afford the insurance premium [27].

### 1.2. Conceptual Framework

The Andersen’s Behavioral Health Model of Health Service Use has been broadly used as a conceptual framework to explain the utilization of health services [28], including outpatient visits. The model postulates that predisposing characteristics (e.g., age, gender, marital status, social structure, and health beliefs), enabling factors (e.g., income, education, health insurance, family, and community), and need factors (e.g., health, functional limitations, and illness) influence the outpatient visits [29,30,31]. Of particular interest in the current investigation are the roles of different health insurance plans and city of residence in outpatient visits among older adults living alone in urban China. Health insurance and city of residence are both enabling factors as they directly affect healthcare resources, social welfare, and services (e.g., escort of older adults), which either facilitate or impede outpatient visits.

### 1.3. Health Insurance and Outpatient Visits

Health insurance increases outpatient visits and improves equity in healthcare among older adults compared to the uninsured group [32,33]; however, few studies examine the associations between different health insurance plans and outpatient visits. The reimbursement ratio, premium contributions, and deductibles vary across different health insurance plans; therefore, it is likely that their association with outpatient visits may differ [21]. Public medical insurance consists of free medical treatments (the cost reimbursement ratio is 90–95%) for outpatient visits, and participants are exempted from premium contributions. Urban employee basic medical insurance is compulsory. Retired workers are exempted from premium contributions when reaching the maximum time period (20–30 years), and they can use individual medical savings accounts for outpatient visits treatments (the reimbursement ratio is 60–75%), with lower deductibles. Urban resident basic medical insurance is voluntary insurance. Participants need to pay premium contributions every year (nearly 200 RMB, equivalent to 30 dollars), and they cannot be reimbursed for outpatient services treatments (only those living in a few big cities, such as Shanghai and Guangzhou, can be reimbursed; the reimbursement ratio is 50–65%) [34]. The financing level of urban employee basic medical insurance is nearly 10 times higher than urban resident basic medical insurance [35], which led the reimbursement ratio of urban employee basic medical insurance (66.8%) to be higher than urban resident basic medical insurance (49.8%) [19]. Thus, the higher the reimbursement ratio of health insurance plan is, the more likely to have outpatient visits among older adults living alone.

### 1.4. City of Residence and Outpatient Visits

Fundamentally, the use of outpatient visits among older adults living alone is not only dependent on health insurance plans but also on where they live (city of residence). Outpatient visits have geographic differences because the geographic location may influence older adults’ choice of receiving healthcare [36], but few researchers examined the association between city of residence and outpatient visits. As economic development and medical resources are different, urban community services and welfare that older adults depend on for health care are also different [37]. Since the start of economic reform in 1978, China’s gap in economic development across cities has grown due to public infrastructure, geographic locations, natural resources, and agglomeration economies. Consequently, the city gaps in economic development result in significant city health inequalities in China [38]. Due to unbalanced development and financing of health insurance among cities, the distribution of services, particularly outpatient care, varies from city to city. It is widely admitted that gross domestic product (GDP) is an important indicator of the economic development of cities. Cities in less developed areas (e.g., western region) generally have lower GDPs, fewer medical resources, and lower levels of health care supply, and thus, may provide fewer choices in outpatient visits compared to the eastern ones [39,40]. Hence, it is important to analyze the differences in outpatient visits among older adults living alone in different cities of China.

### 1.5. Current Study

In summary, this study aimed to examine the association between health insurance, city of residence, and outpatient visits among older adults living alone in Chinese urban areas. Based on the Andersen’s behavioral health model and existing literature, we hypothesize that: (1) older adults living alone with health insurance plans of higher outpatient cost reimbursement ratio are more likely to use outpatient services, and (2) older adults living alone in a more developed city with higher GDPs, more healthcare resources, and better health insurance policies are more likely to use outpatient services.

## 2. Materials and Methods

### 2.1. Participants

The data was derived from “Survey on Older Adults Aged 70 and Above Living Alone in Urban China,” which was a unique dataset of older adults living alone collected from 2013 to 2015 in five cities. The five cities were chosen to reflect the diversity of economic development, medical resources, health policies, and communities among older adults living in urban China. Shanghai (East), Guangzhou (South), and Dalian (Northeast) are typical cities in the Eastern Chinese coastal region, while Chengdu (Southwest) and Hohhot (North) are representative inland cities in China [41]. The largest and most developed city, as well as being a leader in health reform (e.g., coverage of health insurance, community health reform, and chronic disease control), is Shanghai, followed by Guangzhou, Chengdu, Dalian, and Hohhot. Therefore, these five cities are representative cities in their regions and suitable for examining outpatient visits among older adults in urban China. 

For the study, 3363 respondents were recruited through a multi-stage sampling method from 72 communities, 36 streets, and 20 districts within five big cities of China. First, four districts were randomly selected from each city. Second, two streets (one in Shanghai) were randomly selected from each district. Third, two communities were randomly selected from each street. Fourth, 50 respondents (100 in Shanghai) were selected from each community. If the number of respondents in the community was less than the requirements, all qualified individuals were enrolled in accordance with the cluster sampling requirements. Trained interviewers conducted face-to-face interviews. The eligibility of respondents in this study includes: (1) 70 years and older, (2) living in local communities, (3) living alone for more than 6 months, and (4) capable of communication. Of the 3363 respondents, 506 lived in Chengdu, 659 lived in Hohhot, 819 lived in Dalian, 605 lived in Guangzhou, and 774 lived in Shanghai. After deleting missing values in the dependent variable (*n* = 80) and the other variables (*n* = 110), 3173 individuals were included in the final sample of the study. This study exempted from ethics approval, as it was an analysis of secondary data, and the participants provided informed consent prior to the data collection. 

### 2.2. Measures

#### 2.2.1. Outpatient Visits

In this study, outpatient visits were measured by a single question: “In the last 3 months, have you utilized any outpatient visits (examinations, consultations, dispensing/prescription of medication, infusion, preventive screenings, dental visits, or traditional healing)?” The responses were coded as binary variables (0 = no, 1 = yes).

#### 2.2.2. Health Insurance and City of Residence

The explanatory variables were health insurance and city of residence. The health insurance variables were measured by three dummy variables: urban resident basic medical insurance (0 = no, 1 = yes), urban employee basic medical insurance (0 = no, 1 = yes), and public medical insurance (0 = no, 1 = yes), with no health insurance as the reference category. The city of residence was represented by four dummy variables Hohhot (0 = no, 1 = yes), Guangzhou (0 = no, 1 = yes), Dalian (0 = no, 1 = yes), and Shanghai (0 = no, 1 = yes), with Chengdu as the reference category, as its economic development (GDP) and related medical resources are the most moderate of the five cities [42].

#### 2.2.3. Covariate Variables

We also included predisposing characteristics, enabling factors, and need factors as covariate variables based on the Andersen’s behavioral health model. The predisposing characteristics included gender (0 = female, 1 = male), marital status (0 = unmarried, 1 = married), education (0 = illiterate, 1 = primary school or higher), and age. Age was measured in years. 

The enabling factors included self-reported financial status and the number of children. Self-reported financial status was a five-point Likert scale (1 = very poor, 3 = fair, and 5 = very good), with higher scores meaning good economic status. The number of children was a continuous variable, which included both biological children and adopted children, foster children, or stepchildren.

The need factors included self-rated health, functional limitations, and the number of chronic diseases. Self-rated health status was coded on a five-point Likert scale (from 1 = very poor to 5 = very good), with higher values indicating better health status. Because the variable of self-rated functional limitations was not normally distributed, with a high percentage having no functional limitations, we used coding (0 = no functional limitations, 1 = some functional limitations) to present the functional limitations. Chronic diseases diagnosed by a doctor, including hypertension, chronic lung diseases, heart disease, cancer or malignant tumor, stroke, diabetes or high blood sugar, liver disease, kidney disease, arthritis or rheumatism, and psychiatric problems (depression, memory-related disease, and Alzheimer’s), were coded as binary variables (0 = no, 1 = yes). We summed up the 10 items of chronic diseases. The number of chronic diseases ranges from 0 to 10, with a higher score indicating a greater number of chronic diseases.

### 2.3. Statistical Analysis

Continuous variables were expressed as the mean and standard deviation (SD), and categorical variables were expressed as frequencies (%) to describe the respondent’s characteristics. Pearson’s Chi-square tests were used to compare differences between outpatient visits and no outpatient visits. Logistic regression models (95% CI) were used to examine the association between health insurance, city of residence, and outpatient visits among respondents. Model 1 included health insurance and city of residence, controlling the predisposing, enabling, and need factors. In Model 2, we added the number of chronic diseases to Model 1. We also assessed possible multicollinearity among variables and found that all variance inflation factors were 1.18 (less than 3 [43]). All analyses were conducted using Stata/MP 14.0 (StataCorp LP, College Station TX, USA).

## 3. Results

### 3.1. Socio-Demographic Characteristics of the Respondents

Table 1 presents the characteristics of the 3173 respondents. Fifty two percent of the respondents had urban employee basic medical insurance, 26.19% had urban resident basic medical insurance, 4.38% had public medical insurance, and 17.11% did not have any health insurance. Sixteen percent of the respondents lived in Chengdu, 17.18% in Hohhot, 18.44% in Guangzhou, 24.46% in Dalian, and 24.08% in Shanghai. The predisposing characteristics showed that nearly 70% of the respondents were female, and 82.10% were unmarried. Seventy four percent of the respondents had an education level of primary school or higher. The average age of the respondents was 79 years old, ranging from 70 to 107. The enabling factors showed that the average self-reported financial status of respondents was 3.11, ranging from 1 to 5. The average number of children was 2.66, ranging from 0 to 9. The need factors showed that the respondent’s average self-rated health was 3.0, ranging from 1 to 5. Nearly one out of four respondents had some functional limitations (24.05%). The average number of chronic diseases of the respondents was 1.85, ranging from 0 to 10.

### 3.2. Health Insurance and City of Residence by Outpatient Visits

Table 2 shows that only 39.23% of respondents who were uninsured had outpatient visits, while over 50% of respondents with health insurance had outpatient visits. Respondents with public medical insurance (57.55%) were more likely to have outpatient visits than those with urban employee basic medical insurance (55.18%) and urban resident basic medical insurance (52.83%). There were significant differences in outpatient visits among four different health insurance groups of respondents. As for the city of residence, respondents who lived in Guangzhou (67.52%) and Shanghai (67.54%) were more likely to have outpatient visits than those who lived in Chengdu (52.09%), Dalian (36.21%), and Hohhot (35.60%). There were also significant differences in the rate of outpatient visits across the five cities.

### 3.3. Logistic Regression Model

Table 3 reports the association between different types of health insurance plans, city of residence, and outpatient visits. In Model 1, respondents who had urban resident basic medical insurance (OR 1.327, 95% CI 1.026–1.715), urban employee basic medical insurance (OR 1.394, 95% CI 1.098–1.771), and public medical insurance (OR 2.060, 95% CI 1.332–3.186) were more likely to use outpatient visits than those without any health insurance. Model 2 added the number of chronic diseases as a covariate variable. Respondents who had public medical insurance (OR 1.752, 95% CI 1.121–2.739) were more likely to have outpatient visits than those without any health insurance. However, there were no significant differences across other types of health insurance plans, after controlling the number of chronic diseases. Additionally, city of residence was associated with outpatient visits. Respondents who lived in Shanghai (OR 2.402, 95% CI 1.848–3.123) and Guangzhou (OR 2.450, 95% CI 1.857–3.232) were more likely to have outpatient visits than Chengdu, whereas those who lived in the city of Dalian (OR 0.560, 95% CI 0.431–0.728) and Hohhot (OR 0.331, 95% CI 0.244–0.447) were less likely to have outpatient visits. Other factors affecting outpatient visits were listed as follows: respondents with older age and better self-reported health status were less likely to have outpatient visits, while respondents with more children, some functional limitations, and more chronic diseases were more likely to have outpatient visits. 

## 4. Discussion

This study examined the association between health insurance, city of residence, and outpatient visits among older adults living alone in urban areas of China. We found that outpatient visits were associated with different types of health insurance plans, and different cities of residence. Respondents with health insurance of higher reimbursement ratio were more likely to have outpatient visits. However, only public medical insurance had a statistically significant association after controlling the number of chronic diseases. In addition, respondents living in a more developed city were more likely to have outpatient visits. Both hypotheses were supported. 

First, we found that respondents with health insurance were more likely to have outpatient visits than those without any health insurance. This finding is consistent with previous studies, which found that basic health insurance improves outpatient visits for older adults [44]. This is possible because older adults with some health insurance pay more attention to their health status as they have the ability to pay for health care than those without any health insurance [24]. Although health insurance is supposed to be universally covered in urban areas, 17.11% of respondents were still uninsured. Thus, efforts are needed to ensure all older adults are covered by health insurance in China in order to improve health and well-being of this vulnerable population. 

Second, respondents with public medical insurance are more likely to use outpatient visits than those with other types of health insurance plans. This may mainly be because of the differences in the policy design of outpatient reimbursements in different health insurance plans in China in terms of reimbursement ratio, insurance coverage, and deductibles. Furthermore, health insurance plans remain fragmented; thus, different types of health insurance plans would cause health-equity issues related to outpatient visits [45,46]. A reform to integrate different health insurance plans is one of the key strategies addressing issues of inequity in outpatient visits caused by a fragmented health system in China. In recent years, the central government has made efforts to narrow the gap between different population groups in terms of health insurance, and some local governments have taken measures to combine the urban employee basic medical insurance, urban resident basic medical insurance, and public medical insurance into the Uniform Social Basic Medical Insurance [19,24]. Future studies should focus on exploring the integration reform of different health insurance plans to outpatient visits of older adults in Chinese urban areas.

Third, this study also reveals that after controlling the number of chronic diseases, only respondents with public medical insurance are more likely to have outpatient visits than the uninsured. This result is different from the previous study, which showed that older adults with employee basic medical insurance and urban resident basic medical insurance are more likely to have outpatient visits than the ones without health insurance when controlling the number of chronic diseases [15]. One possible explanation is due to different study populations between our current study and previous studies. Nearly 90% of respondents have chronic diseases in this sample, which reflects greater needs of health care than the general population. However, only those with public medical insurance can get reimbursement for most chronic disease treatments in outpatient visits. Those with urban employee basic medical insurance and urban resident basic medical insurance get very limited or no reimbursement for chronic disease treatments in outpatient visits, because the Chinese government implemented inpatient treatment-oriented benefits packages, neglecting prevention and outpatient services [19]. Such arrangements may lead to delayed chronic disease treatment among older adults with limited financial resources; more importantly, it could increase unnecessary hospitalization and increase healthcare cost. On the other hand, older adults living alone with chronic diseases who qualify and yet do not apply for these programs are less likely to use outpatient visits, as they are unaware that they qualify or they are unable to complete the complex paperwork [7]. In addition, self-treatment, such as the use of over the counter medication, is negatively associated with outpatient visits for older adults with chronic diseases in China [47]. Future studies should shift focus on accessibility to primary health care to chronic disease prevention, management, and treatment, in order to improve the effectiveness of healthcare system in mitigating these inequalities, with special consideration given to this group of older adults.

Additionally, the city of residence was also associated with outpatient visits. Respondents living in the east and south coast of China (e.g., Shanghai and Guangzhou) are more likely to have outpatient visits than those living in the west, north, and northeast inland of China (e.g., Chengdu, Dalian, and Hohhot). This result is in line with a Canadian study that showed that area variation is the most important source of income-related health inequality, with income-related inequities in outpatient visits being mostly driven by differences between cities [48]. The cities in eastern and central regions have better health spatial accessibility, while in the western regions there is more limited spatial accessibility [49]. Moreover, this imbalance in outpatient visits can also be explained by different levels of medical resources and health insurance policies in different cities of residence [40]. The urban resident basic medical insurance policy in Shanghai and Guangzhou covers general outpatient visits treatments, but the ones in Chengdu, Dalian, and Hohhot do not cover general outpatient visits treatments. Furthermore, community services and welfare, which many older adults living alone depend on, vary across different cities. Shanghai has better community services or long-term care for older adults living alone than the other cities [50]. To adequately address disparities in outpatient visits, the city of residence needs to be incorporated into discussions. Policymakers should take action to improve access to outpatient visits, health resources, and health insurance policies across cities.

This study has several limitations. First, the data is cross-sectional, which restricts drawing causal conclusions about the relationship between health insurance, city of residence, and outpatient visits. Second, the sample of this study is limited to urban residents, and thus, the finding of this study cannot be generalized to the rural areas. The results could be very different given the different structures of the health care system and health care delivery system between urban and rural areas of China. Third, recall biases are inevitable in questionnaire-based surveys, especially those pertaining to health services use. Future studies could consider using electronic health records, which are more accurate. Finally, given greater attention to the health care reform by the government in China, changes may occur in health insurance policy after the period from 2013 to 2015. Therefore, we should be careful to make results and conclusions. However, by focusing on older adults living alone, the increasing population with greater needs and difficulties in outpatient service utilization, this study sheds important light on the importance of integrating health insurance plans under the continuous health care reform in China.

## 5. Conclusions

In conclusion, this study demonstrates that health insurance and the city of residence are associated with outpatient visits among older adults living alone in China. Older adults living alone who have better health insurance in terms of higher reimbursement ratio and are living in more developed cities are more likely to have outpatient visits than their counterparts. Our findings may contribute to understanding more about inequity in outpatient visits among older adults living alone in urban China, as well as promote health and well-being among this vulnerable group of older adults. Ensuring the equity of access to quality health care remains a great challenge for China and other low-and middle-income countries. Future comparison studies between China and other countries should be conducted in this area to improve access to and quality of healthcare in older adults globally.

## Figures and Tables

**Table 1:** Sample characteristics (*n* = 3173).

Variables	*n* (%)	M (SD)
**Health insurance**		
No health insurance	543 (17.11)	
Urban resident medical insurance	831 (26.19)	
Urban employee medical insurance	1660 (52.32)	
Public medical insurance	139 (4.38)	
**City** **of residence**		
Chengdu	503 (15.85)	
Hohhot	545 (17.18)	
Guangzhou	585 (18.44)	
Dalian	776 (24.46)	
Shanghai	764 (24.08)	
**P** **redisposing characteristics**		
Gender		
Female	2198 (69.27)	
Male	975 (30.73)	
Age		78.978 (5.556) [70, 107]
Marital status		
Unmarried	2605 (82.10)	
Married	568 (17.90)	
Education		
Illiterate	812 (25.59)	
Primary school or higher	2361 (74.41)	
**E** **nabling factors**		
Self-reported financial status		3.113 (0.843) [1, 5]
Number of children		2.657 (1.403) [0, 9]
**Need factors**		
Self-rated health status		3.003 (1.068) [1, 5]
Functional limitations		
No functional limitations	2410 (75.95)	
Some functional limitations	763 (24.05)	
Number of chronic diseases		1.845 (1.215) [0, 10]

**Table 2:** Health insurance and city of residence by outpatient visits.

Variables	Outpatient Visits *n* (%)		*p*-Value
	Yes (*n* = 1648)	No (*n* = 1525)	
**Health insurance**			<0.001
No health insurance	213 (39.23)	330 (60.77)	
Urban resident basic medical insurance	439 (52.83)	392 (47.17)	
Urban employee basic medical insurance	916 (55.18)	744 (44.82)	
Public medical insurance	80 (57.55)	59 (42.45)	
**City** **of residence**			<0.001
Chengdu	262 (52.09)	241 (47.91)	
Hohhot	194 (35.60)	351 (64.40)	
Guangzhou	395 (67.52)	190 (32.48)	
Dalian	281 (36.21)	495 (63.79)	
Shanghai	516 (67. 54)	248 (32.46)	

**Table 3:** Logistic regression models for factors associated with outpatient visits.

Variables	Model 1	Model 2
	OR (95% CI)	OR (95% CI)
**Health insurance (ref = no insurance)**		
Urban resident basic medical insurance	1.327 (1.026–1.715)	1.226 (0.942–1.595)
Urban employee basic medical insurance	1.394 (1.098–1.771)	1.212 (0.948–1.549)
Public medical insurance	2.060 (1.332–3.186)	1.752 (1.121–2.739)
**City** **of residence (ref = Chengdu)**		
Hohhot	0.434 (0.325–0.579)	0.331 (0.244–0.447)
Guangzhou	2.423 (1.847–3.179)	2.450 (1.857–3.232)
Dalian	0.599 (0.464–0.773)	0.560 (0.431–0.728)
Shanghai	2.396 (1.855–3.094)	2.402 (1.848–3.123)
**Predisposing characteristics**		
Male (ref = female)	0.825 (0.689–0.987)	0.866 (0.721–1.040)
Age	0.983 (0.967–0.999)	0.982 (0.966–0.998)
Married (ref = Unmarried)	1.142 (0.920–1.417)	1.107 (0.889–1.378)
Primary school or higher (ref = Illiterate)	1.228 (1.012–1.490)	1.207 (0.990–1.472)
**Enabling factors**		
Self-reported financial status	0.932 (0.839–1.034)	0.944 (0.849–1.050)
Number of children	1.101 (1.036–1.169)	1.090 (1.025–1.159)
**Need factors**		
Self-rated health status	0.494 (0.452–0.540)	0.596 (0.542–0.655)
Some functional limitations (ref = no)	1.351 (1.097–1.664)	1.284 (1.038–1.588)
Number of chronic diseases		1.572 (1.448–1.708)
R^2^	0.1606	0.1893
Log likelihood	−1844.2034	−1781.1909
